# Stabilization of primary cilia reduces abortive cell cycle re-entry to protect injured adult CNS neurons from apoptosis

**DOI:** 10.1371/journal.pone.0220056

**Published:** 2019-08-01

**Authors:** Brian K. A. Choi, Philippe M. D’Onofrio, Alireza P. Shabanzadeh, Paulo D. Koeberle

**Affiliations:** 1 Department of Surgery, Division of Anatomy, University of Toronto, Toronto, ON, Canada; 2 Graduate Department of Rehabilitation Sciences, University of Toronto, Toronto, ON, Canada; 3 Krembil Research Institute, University of Toronto, Toronto, ON, Canada; Universidade Federal do Rio de Janeiro, BRAZIL

## Abstract

Abortive cell cycle (ACC) re-entry of apoptotic neurons is a recently characterized phenomenon that occurs after central nervous system (CNS) injury or over the course of CNS disease. Consequently, inhibiting cell cycle progression is neuroprotective in numerous CNS pathology models. Primary cilia are ubiquitous, centriole-based cellular organelles that prevent cell cycling, but their ability to modulate abortive cell cycle has not been described. Here, we show that neuronal cilia are ablated *in-vitro* and *in-vivo* following injury by hypoxia or optic nerve transection (ONT), respectively. Furthermore, forced cilia resorption sensitized neurons to these injuries and enhanced cell death. In contrast, pharmacological inhibition or shRNA knockdown of the proteins that disassemble the cilia increased neuron survival and decreased the phosphorylation of retinoblastoma (Rb), a master switch for cell cycle re-entry. Our findings show that the stabilization of neuronal primary cilia inhibits, at least transiently, apoptotic cell cycling, which has implications for future therapeutic strategies that halt or slow the progression of neurodegenerative diseases and acute CNS injuries.

## Introduction

Fatal cell cycle re-entry by neurons, known as abortive cell cycling (ACC), has been observed in a number of CNS diseases such as Alzheimer’s and Parkinson’s disease [1,2] or insults that include stroke [3] and traumatic brain injury (TBI) [4]. Injured neurons exhibit DNA replication and elevation of cell cycle markers such as phosphorylated retinoblastoma (pRb) [5]. Inhibiting cell cycle progression, through cyclin dependent kinase (CDK) inhibitors or knockout of mitotic transcription factors, has been neuroprotective in a number of CNS injury models, implicating ACC as a critical process in neuronal apoptosis [5–8].

Primary cilia are solitary projections of the cell body that suppress cell cycle by sequestering centrioles at the cell membrane [9,10]. Each eukaryotic cell has two centrioles (mother and daughter) that are bound together forming the centrosome [11]. Cilia have a microtubule backbone (axoneme) anchored to a modified mother centriole called the basal body [9]. Upon cell cycle entry, the axoneme is disassembled to free the centrosome for cell division. This occurs through the activity of histone deacetylase 6 (HDAC6), which deacetylates axoneme tubulin [12]. HDAC6 is phosphorylated by Aurora Kinase A (AurA), which itself is activated by Human Enhancer of Filamentation 1 (HEF1) and Trichoplein (TCHP) [12,13]. Furthermore, Polo-like kinase 1 (PLK1) stabilizes HEF1 [14] and activates HDAC6 [15] and Kinesin family member 2a (Kif2a) [16]. Kif2a associates with the basal body to promote cytoplasmic microtubule depolymerization [16] while NudE homolog 1 (NDE1) assists in cilia retraction by sequestering dynein. Dynein regulates intraflagellar transport (IFT) that is required for cilia assembly and maintenance [17,18]. Tellingly, rapidly dividing cells have a reduced frequency of cilia and increased levels of cilia disassembly proteins [19,20]. Conversely, suppression of cilia disassembly proteins enhances ciliogenesis and inhibits proliferation [17,21,22]. As the cilia are nearly ubiquitous in neurons and act as a cell cycle brake, we sought to investigate if ciliary integrity plays a role in ACC and can be manipulated to halt its progression.

Here, we report that adult CNS neuron injury results in the shortening and loss of primary cilia. Furthermore, the stabilization of primary cilia, by inhibiting disassembly mechanisms, is neuroprotective and reduces cell cycle re-entry. In contrast, truncating cilia by pharmacological inhibition or knockdown of IFT components enhanced neuron apoptosis after injury. These findings establish a novel role for primary cilia in regulating cell cycle re-entry and concomitant apoptosis in mature mammalian CNS neurons.

## Materials and methods

Animal Care Committee at the University of Toronto approved this research. Protocol Numbers: 20011691, 2001699. Animals (Sprague Dawley Rats) were anesthetized using isofluorane.

### Primary cortical neurons

Primary cortical neurons were isolated from P1 Sprague Dawley rats. The pups were fully anesthetized in a chamber with 4% isoflurane to reduce suffering before they were euthanized by decapitation with large surgical scissors. Cortices were dissected and freed of meninges in ice cold, calcium and magnesium free Hank’s balanced salt solution and dissociated in 0.05% trypsin for 15 minutes at 37°C. Trypsinization was terminated by adding GIBCO Dulbecco’s Modified Eagle Medium (DMEM) (ThermoFisher Scientific, Waltham, MA) containing 10% fetal bovine serum (FBS) (ThermoFisher Scientific, Waltham, MA) at double the volume of the trypsin. Next, the cortices were triturated using a fire polished Pasteur pipette. The dissociated neurons were then run through a 40 um cell strainer (ThermoFisher Scientific, Waltham, MA) and centrifuged at 900 RPM for 4 minutes. The resulting pellet was resuspended in Neurobasal media (ThermoFisher Scientific, Waltham, MA) containing 2% B27 (ThermoFisher Scientific, Waltham, MA), 1% glutamine (ThermoFisher Scientific, Waltham, MA), and 1% penicillin-streptomycin (P/S) (Sigma-Aldrich Canada Corp., Oakville, ON) and plated at a density of 50 000 cells/cm^2^ on 30,000–70,000 Da Poly-D-Lysine (PDL) (Sigma-Aldrich Corp., Oakville, ON) coated 96 well plates (Sarstedt Inc., Saint-Léonard, QC). Cells were kept in a humidified incubator at 37°C containing 95% air and 5% CO2. At 3 days *in-vitro* (DIV), half of the culture media was replaced to add Ara-c at a final concentration of 1 uM to inhibit the growth of glial cells. At 5 DIV half the culture media was changed with fresh media. Cultures were used after 7 DIV.

### Cell lines

N1E-115 cells (CRL-2263, ATCC, Manassas, VA) were purchased from the manufacturer and grown in T-75 flask (Sarstedt Inc., Saint-Léonard, QC) with DMEM/F12 (ThermoFisher Scientific, Waltham, MA) supplemented with 10% FBS, 5% horse serum (ThermoFisher Scientific, Waltham, MA), and 1% P/S in incubators at 37°C with 95% air and 5% CO2. Cells were passaged weekly at 70–80% confluency. N1E-115 cells were differentiated by culturing on PDL coated plates at a density of 15 000 cells/cm^2^ in DMEM with 1% FBS and 1.5% dimethyl sulfoxide at 37°C containing 95% air and 5% CO2. Cells were used 5–7 DIV. HT22 cells (SCC129, Millipore Sigma, Burlington, MA) were kindly donated from Dr. Jeremy Sivak’s lab and grown in T-75 flasks with DMEM supplemented with 10% FBS and 1% P/S in incubators at 37°C with 95% air and 5% CO2. Cells were passaged weekly at 70–80% confluency. HT22 cells were differentiated by plating on PDL coated plates in complete growth medium which was replaced with Neurobasal containing 1% N-2 supplement (ThermoFisher Scientific, Waltham, MA), 1% glutamine, and 1% P/S for 24 hours before use. RPE1 cells (CRL-4000, ATCC, Manassas, VA) were kindly donated from Dr. William Trimble’s lab and grown in T-75 flasks in DMEM/F12 containing 10% FBS and 1% P/S in incubators at 37°C with 95% air and 5% CO2. The cells were passaged weekly at 70–80% confluency. For experimentation, RPE1 cells were plated on PDL coated plates in complete growth medium. When the cultures became confluent the growth media was replaced with serum-free DMEM with 1% P/S for 24 hours to induce ciliogenesis before use.

### Etoposide induced apoptosis and proliferation assay

Cells were pre-treated with either Tubastatin A (Selleckchem, Houston, TX, Catalog No. S8049) or Aurora Inhibitor I (Selleckchem, Houston, TX, Catalog No. S1451) for 1 hour before injury. Apoptosis was induced by 24 hours of 50 μM of etoposide (Sigma-Aldrich Canada Corp., Oakville, ON) treatment. Cell survival was assessed by incubating cultures with 0.5mg/mL MTT (Sigma-Aldrich Canada Corp., Oakville, ON) at 10% of culture volume for 4 hours at 37°C. The resulting MTT crystals were dissolved with DMSO for 1 minute with gentle shaking. Similarly, WST-8 proliferation assay reagent (Sigma-Aldrich Corp., Oakville, ON) was added to the cell culture media at 10% of the culture volume for 4 hours at 37°C. A Tecan Fluoroskan microplate reader (Männedorf, Switzerland) was used to measure optical density of the solution at a wavelength of 570 nm, with the reference wavelength at 620 nm, or 450 nm for the MTT and the WST-8 assay respectively.

### Optic nerve transection

All animals were cared for according to the Canadian Council on animal care. Adult female albino Sprague-Dawley rats (Charles River Laboratories, Senneville, QC), free of common pathogens, were used in all experiments. These were kept at the University of Toronto animal facilities, in a pathogen-controlled environment, housed in standard cages, on a 12 hour light/dark cycle. Food and water were provided as needed.

For surgical procedures, animals were individually anesthetized with isoflurane (2%; 0.8% L/min oxygen flow rate) and placed in a stereotaxic frame with anesthesia delivered through a gas mask to maintain sedation. Tears Naturale lubricant (Alcon Canada Inc., Mississauga, ON) was applied to the cornea to prevent desiccation during surgery. To minimize pain and aid in postoperative recovery all animals were given one pre-operative injection of ketoprofen (5 mg/mL, dosage for rats; 0.1 mL/100 g body weight). To access the optic nerve, an incision was made in the tissue covering the superior border of the orbital bone, followed by dissection of the superior orbital contents and retraction of the overlying rectus muscles. The dural sheath surrounding the optic nerve was cut longitudinally to avoid damaging the blood vessels supplying the retina. For optic nerve transection, the nerve was carefully lifted from the meningeal sheath, and cut within 2 mm of the back of the eye. Orbital contents were then returned to their original location, and the initial incision was closed. Following surgery, the animals were placed in a recovery cage under a heat lamp. The following day, the rats were given an intraperitoneal injection of ketoprofen (5 mg/mL, dosage for rats; 0.1 mL/100 g body weight) to minimize postoperative discomfort. Ophthalmic eye ointment was again applied to the cornea to ensure that it remained hydrated during the recovery period.

### Intraocular injections

As RGC apoptosis commences 3–4 days after injury [23,24], there is a window for treatment delivery to target the apoptotic machinery. 4 uL of Tubastatin A or Aurora A Inhibitor I dissolved in DMSO were injected at 3 and 10 days following surgery. To deliver the intraocular injections, animals were anesthetized with isoflurane and placed in a stereotaxic frame under continued anesthesia (2% isoflurane; 0.8% L/min oxygen flow rate) delivered through a gas mask. A pulled glass micropipette attached to a 10 μL Hamilton syringe via a hydraulic coupling was used for the intraocular injections. The injection was made into the vitreous chamber of the eye, posterior to the limbus, with care taken to prevent damage to the lens or the anterior structures of the eye. After injecting the appropriate solution, the pipet was held in place for 5 seconds in order to prevent reflux, and then carefully withdrawn from the eye. Injections were carried out under a surgical microscope to visualize pipet entry into the appropriate location and to confirm delivery of the solution into the vitreous chamber.

### DNA construct, AAV2 packaging, and neuronal infection

The AAV2 vectors used to express EGFP and shRNAs were constructed and packaged by VectorBuilder (Cyagen Biosciences, Santa Clara, CA). The shRNA sequences are listed below. All shRNA expression was driven by a U6 promoter while the EGFP tracer was driven by a phosphoglycerate kinase (PGK) promoter. The vector sequences are as follows:

IFT88: 5’-TTGACGAAGACGATAAATATA CTCGAG TATATTTATCGTCTTCGTCAA-3’

Kif2a: 5’-CCTGGGATGTGATGGTTATTT CTCGAG AAATAACCATCACATCCCAGG-3’

HEF1: 5’-GAAAGGACTGGTTGAATAATT CTCGAG AATTATTCAACCAGTCCTTTC-3’

PLK1: 5’-CCTCTCACAGTCCTTAATAAA CTCGAG TTTATTAAGGACTGTGAGAGG-3’

NDE1 5’-ACTTCCAGCTGAAGCTATATT CTCGAG AATATAGCTTCAGCTGGAAGT-3’

Control: 5’-CCTAAGGTTAAGTCGCCCTCG CTCGAG CGAGGGCGACTTAACCTTAGG-3’

TCHP: 5’-GAGGTCACACACTGCTTTATA CTCGAG TATAAAGCAGTGTGTGACCTC-3’

RGCs were infected with the AAV2 by intraocular injection of 4 uL of stock virus titer (>10^11^ GC) and 1 uL of 0.0002% pronase E (Sigma-Aldrich Corp., Oakville, ON) to digest the retinal inner limiting membrane and improve viral penetration of the GCL. All injections were carried out at the U of T animal facility BSL2 labs by staff trained in biosafety and AAV2 handling. Approximately 3 weeks were given for shRNA expression before optic nerve transection or retinal extraction.

### Western blots

The animals were anesthetized in a chamber containing 4% isoflurane to reduce suffering before euthanasia by cervical dislocation. Anesthetic depth was confirmed by toe pinch. The eyes were enucleated and the retinas were immediately extracted in ice-cold PBS. Retinas were individually sonicated in 400 μL solutions of ice-cold SDS lysis buffer (2% SDS, 0.3% DTT, 10% glycerol in 40 mM Tris-Cl, pH 6.8) prior to denaturing (heated to 90°C for 8 minutes) and removal of cellular debris by centrifugation (12,000 rpm, 10 min, 4°C). Proteins were separated by SDS-PAGE on 4–20% polyacrylamide Bio-Rad TGX gels and then transferred via semi-dry method to a 0.2 μm pore nitrocellulose membrane. Membranes were blocked in 5% milk solution for 1 hour at room temperature. Primary antibodies were dissolved in 1% milk, and blots were incubated in the solution overnight at 4°C on a platform rocker. The secondary antibody was HRP-conjugated, and blots were incubated in secondary for 1 hour at room temperature on a platform rocker. Imaging was performed using a Bio-Rad Fluor-S Max imager (Bio-Rad, Mississauga, ON). Loading was verified by re-probing the blots with antisera directed against GAPDH (1:1000; rabbit polyclonal; Cell Signaling Technology).

### Immunohistochemistry and quantification of RGC survival

#### Flatmounted retinas

The animals were anesthetized in a chamber containing 4% isoflurane to reduce suffering before euthanasia by cervical dislocation. Anesthetic depth was confirmed by toe pinch. The animals were sacrificed at 7 or 14 days after optic nerve transection. The eyes were enucleated and the eyecups, containing the retinas, were fixed in 4% paraformaldehyde (PFA) (Bioshop Canada Inc., Burlington, ON) for 1 hour at room temperature. Next, the retinas were extracted, washed 3x10 min in PBS, and blocked in PBS containing 0.3% Triton X-100 (TPBS), 3% normal donkey serum (NDS) (Sigma-Aldrich Corp., Oakville, ON), and 1% Bovine serum albumin (BSA) (ThermoFisher Scientific, Waltham, MA) for 1 hour. After blocking, the samples were incubated in primary antibodies in blocking solution overnight at 4°C at the concentrations listed below. The samples were washed 3x10 min in PBS and incubated with secondary antibodies conjugated with Cy2 or Cy3 fluorophores (1:500) (Jackson ImmunoResearch Laboratories, Inc., West Grove, PA) overnight at 4°C. Finally, the samples were washed 3x10 min in PBS and flatmounted. The retina was coverslipped in a solution of 50:50 glycerol/PBS and sealed with nail polish.

The following primary antibodies were used:

Rabbit Polyclonal anti-ACIII (Santa Cruz Biotechnology, Dallas, TX, sc-588, 1:250), Goat Polyclonal anti-Brn3a (Santa Cruz Biotechnology, Dallas, TX, sc-31984, 1:250), Mouse Monoclonal anti-AcTub (Santa Cruz Biotechnology, Dallas, TX, sc-23950, 1:100), Mouse Monoclonal anti-pRb (Santa Cruz Biotechnology, Dallas, TX, sc-377528, 1:250), Goat Polyclonal anti-SSTR3 (Santa Cruz Biotechnology, Dallas, TX, sc-11617, 1:250), Mouse Monoclonal anti-NeuN (United States Biological, Salem, MA, N2173, 1:250), Guinea Pig Polyclonal anti-RBPMS (Phosphosolutions, Aurora, CO, 1832-RBPMS, 1:500), Rabbit Polyclonal anti-RBPMS (Phosphosolutions, Aurora, CO, 1830-RBPMS, 1:500), Goat Polyclonal anti-Ki67 (Santa Cruz Biotechnology, Dallas, TX, sc-7844, 1:100),

The retinas were visualized using an Andor iXon 885+ EMCCD camera (Andor Technology, Belfast, Northern Ireland), attached to a Leica DM LFSA microscope (Leica Microsystems, Concord, Canada), with a Sutter Lambda XL light source (Quorum Technologies, Guelph, Canada). To measure RGC density, samples were taken at 3 different eccentricities of retinal quadrants: inner (1/6 of the retinal radius from center), middle (1/2 of the retinal radius from center), outer (5/6 of the retinal radius from center).

#### Transverse sectioned retinas

Animals were given an intracardial perfusion of 4% PFA, and the eye cup was isolated. The eye cup underwent further fixation in 4% PFA for 1 hour at room temperature and washed with PBS (3x15 minutes). Next the eye cup was cryoprotected by immersion in 30% sucrose in PBS until the tissue was no longer buoyant in the sucrose solution (1–2 days). The retina was then sectioned using a Leica CM1950 cryostat microtome. Transverse sections, 14 μm in thickness, were collected onto slides coated with APTEX (Sigma-Aldrich Corp., Oakville, ON), and stored at -20°C until immunostaining was performed.

Immunohistochemistry was performed with antibodies directed against pRb and RBPMS. A water repellent border was drawn around the retinal sections using a pap-pen to concentrate the antibody solution to the samples. Transverse sections of the nerves were incubated overnight at 4°C with primary antibodies diluted in TPBS containing 3% NDS, and 1% BSA. Following the incubation period with primary antibody, sections were rinsed three times in PBS for 15 minutes. This was followed by a 3 hour incubation period with Cy3-conjugated secondary antibody (Jackson ImmunoResearch Laboratories, Inc., West Grove, PA) at room temperature, and three subsequent 15 minute rinses in PBS. The sections were then coverslipped with 50:50 glycerol/PBS. The samples were examined using an AxioObserverZ1 inverted motorized microscope (Carl Zeiss Canada Ltd., Toronto, ON) equipped with a CSU-X1 SDC (Yokogawa Canada Inc., Calgary, AB) with an Axiocam 506 high resolution camera (Carl Zeiss Canada Ltd., Toronto, ON). Variation in tissue depth and focus was accounted for during imaging by capturing an extended depth of field generated by merging the maximum intensity projections of the Z-stack in Zen 2 (Carl Zeiss Canada Ltd., Toronto, ON).

#### Cell culture immunohistochemistry

For immunofluorescence of cell cultures, the cells were grown on glass coverslips pre-coated with (PDL) (Neuvitro, Vancouver, WA) and fixed with 4% PFA for 10 minutes. The coverslips were washed 3x with PBS and permeabilized with TPBS for 10 minutes and blocked with PBS containing 1% BSA and 3% NDS. The coverslips were coated with primary antibodies diluted in blocking buffer at 4°C overnight and was washed 3x with PBS before incubation with secondary antibodies in blocking solution at room temperature for 1 hour. Primary and secondary antibodies were diluted at the same concentrations mentioned above.

#### TUNEL assay

Sectioned retinas were incubated in a solution containing 0.67% terminal deoxynucleotidyl transferase, 0.67% Biotin dUTP, 20% CoCl2 (Roche Diagnostics, Indianapolis, IN) for 2 hours at 37°C. Next, the samples were washed 3x10 mins with 1% PBS tween and incubated in a 1% PBS tween solution with Alexa 555 streptavidin conjugate (1:750, ThermoFisher Scientific, Waltham, MA) for 1 hour in room temperature. After washing 3x10 mins with 1% PBS tween, the samples were cover slipped and sealed in 50:50 glycerol/PBS solution.

### Spinning disc confocal microscopy Z-stacks

To create 3D images of primary cilia for length measurements, an AxioObserverZ1 inverted motorized microscope (Carl Zeiss Canada Ltd., Toronto, ON) equipped with a CSU-X1 SDC (Yokogawa Canada Inc., Calgary, AB) with an Axiocam 506 high resolution camera (Carl Zeiss Canada Ltd., Toronto, ON) at the University of Toronto Microscope Imaging laboratory was used. Similar to the RGC density sampling, images were taken at 3 different eccentricities (inner, middle, outer) of retinal quadrants using a 63x oil objective lens (Carl Zeiss Canada Ltd., Toronto, ON). Z-stacks were generated from the images using Zen 2 software (Carl Zeiss Canada Ltd., Toronto, ON). The Z-stacks were analyzed using Imaris (Bitplane, Belfast, United Kingdom) to obtain cilia lengths.

### Statistical analysis

An unpaired two-tailed Student’s t-test was used to determine statistical significance between two groups. A p value <0.05 was considered statistically significant. The mean ± standard error of the mean (SEM) of each group are displayed in the results. GraphPad InStat was used to conduct the statistical analyses (GraphPad Software Inc., La Jolla, CA). Statistically significant differences between treatments that were used to assess RGC survival after axotomy were calculated by Tukey’s Post hoc tests, following an ANOVA.

## Results

### Sublethal hypoxic stress induces ciliary shortening that is reversed by normoxia

**Fig 1:**
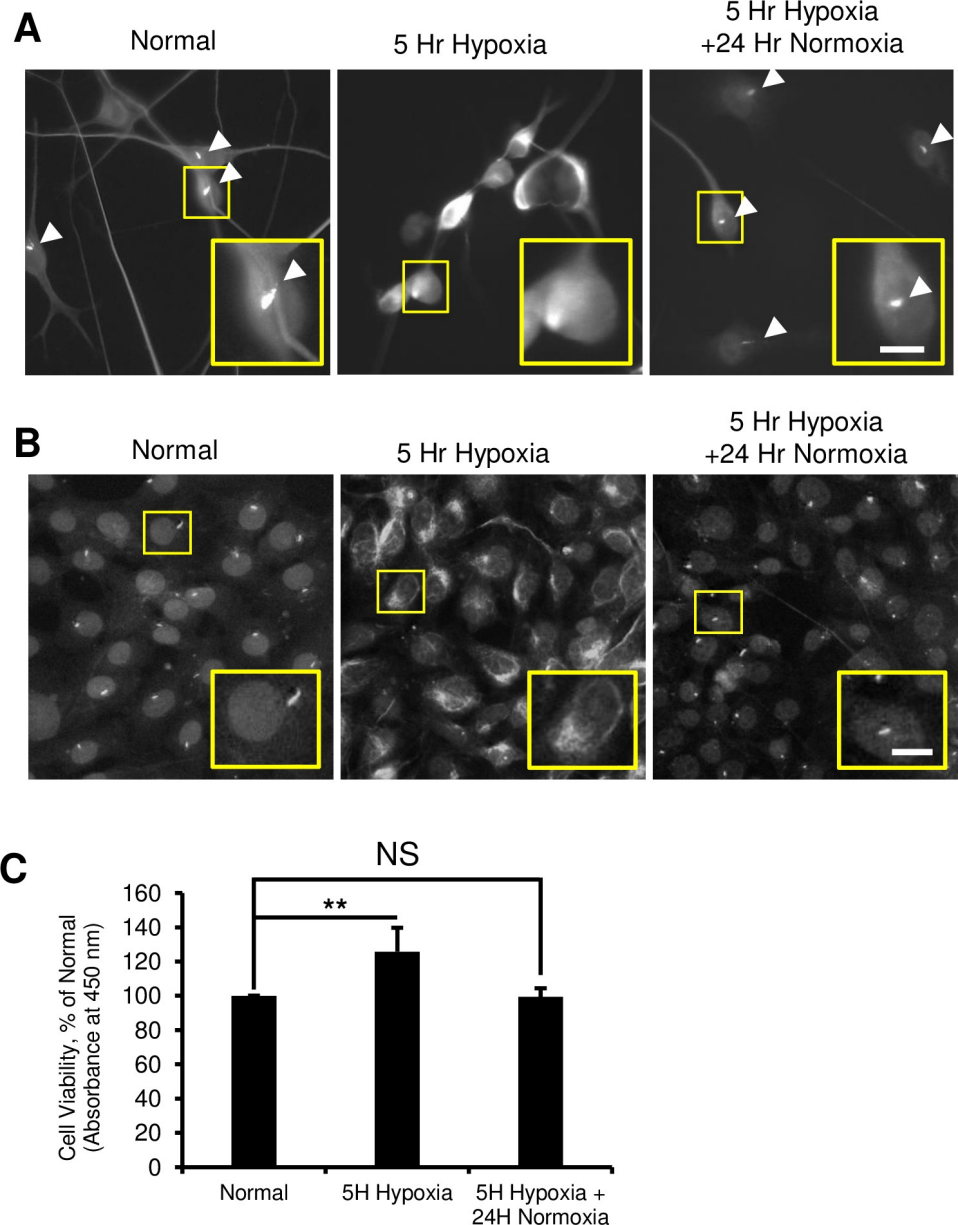
Hypoxia causes reversible neuronal cilia resorption. (A and B) Normal N1E-115 cells (Left A) and HT22 cultures (Left B) exhibited primary cilia (arrowheads in A) demonstrated by immunofluorescence against Actub. Five hours of hypoxia (middle) resulted in the loss of primary cilia and diffuse staining of Actub in both cell lines (Middle panel). Following hypoxia, 24 hours of normoxia returned the expression of primary cilia in both cell lines (Right). N1E-115 Scale bar = 25 μm, HT22 Scale bar = 10 μm. (C) Quantification of N1E-115 cell survival by WST-8 assay showing increased metabolic activity at 5 hours of hypoxia that normalized after 24 hours of normoxia. Data presented as mean ± SEM relative to normal. ** p<0.01.

To investigate if ACC can be modulated by primary cilia, we sought to establish whether apoptotic stimuli alters cilia expression in neurons. Hypoxic injury was utilized because it can be easily controlled and terminated, and has been shown to induce cell cycle re-entry in post-mitotic neurons [25,26]. Differentiated mouse neuroblastoma N1E-115 cultures were exposed to hypoxia for 5 hours then returned to normoxic conditions for an additional 24 hours. Cilia expression was examined by immunofluorescence against the axoneme marker Acetylated Alpha Tubulin (AcTub). Primary cilia were observed in pre-hypoxic normal cell cultures (Fig 1A, Left) but absent at 5 hours following hypoxia, with AcTub dispersed throughout the cytoplasm (Fig 1A, Middle). In contrast, cultures that were returned to normoxic conditions after hypoxia demonstrated a re-expression of primary cilia that were shorter in length compared to pre-hypoxic cells (Fig 1A, Right). We then measured N1E-115 viability and found a significant increase in metabolic activity at 5 hours of hypoxia, concurrent with ciliary retraction, which returned to normal levels after 24 hours of normoxia (Fig 1C, **p<0.01). Next, we replicated these phenotypes in the HT-22 immortalized mouse hippocampal cell line. These cells also demonstrated robust cilia expression (Fig 1B, Left), that disappeared after hypoxia (Fig 1B, Middle). Furthermore, these cultures exhibited the same cytoplasmic dispersal of AcTub that was characteristic of N1E-115 cells after hypoxia. When the hypoxia-exposed cultures were returned to 24 hours of normoxia, cultures similarly re-expressed shortened cilia (Fig 1B, Right). These findings suggest that hypoxic insult causes retraction of the primary cilia, and cells that survive hypoxia re-express cilia after the initial dispersal of AcTub throughout the cytoplasm.

### Primary cilia stability impacts neuron apoptosis *in-vitro*

**Fig 2:**
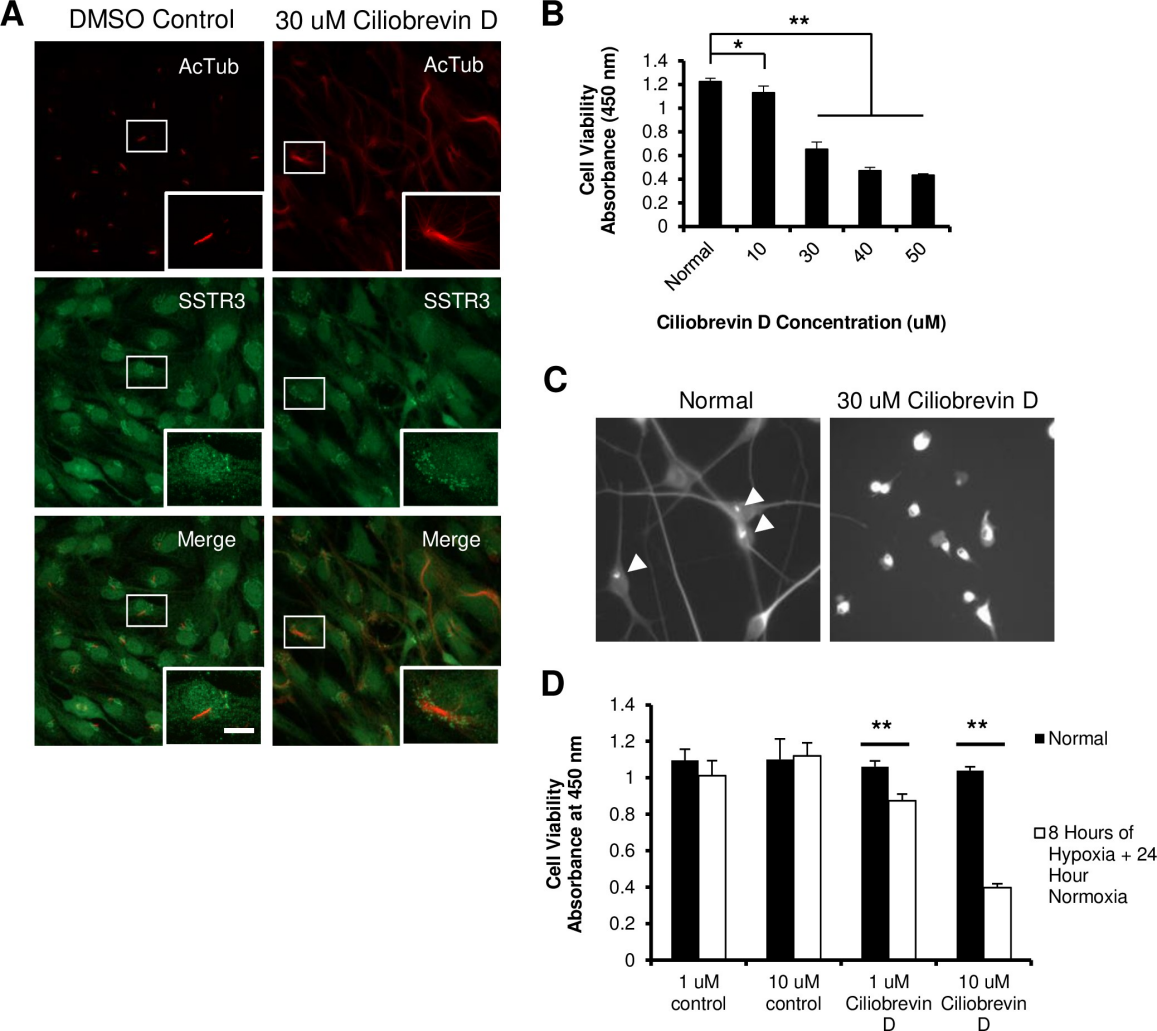
Disruption of IFT ablates cilia and sensitizes N1E-115 cells to hypoxia. (A) Dynein inhibition via 24 hours of Ciliobrevin D treatment disrupted the normal axoneme organization resulting in the loss of primary cilia in RPE1 cells and disarrayed tubulin organization (Right). Green = somatostatin 3 receptor, Red = Acetylated α tubulin. Scale bar = 10 μm. (B) N1E-115 Ciliobrevin D toxicity curve as measured by WST-8 assay (G) (**p<0.01, *p<0.05) showing increasing cell toxicity with higher concentrations. (C) Representative micrographs of immunofluorescence against AcTub in normal N1E-115 cells, to identify primary cilia (arrowhead), compared to a culture treated with 30 μM Ciliobrevin D (Right). Primary cilia were lost and neurites were also ablated. (D) Quantification of N1E-115 survival by WST-8 after hypoxia. Ciliobrevin D treatment increased sensitivity to sublethal hypoxia compared to DMSO control, which had no effect. **p<0.01, *p<0.05. Data presented as mean ± SEM.

After observing the correlation between hypoxic stress and ciliary resorption, we evaluated whether forced disassembly of cilia would impact neuron survival after injury. Given that mutations of IFT components hamper proper ciliogenesis and underlie diseases resulting from cilia malformation [27,28], we inhibited the dynein motor protein to disrupt primary cilia assembly and maintenance. To do so, we treated ciliated Retinal Pigment Epithelial 1 (RPE1) cells with 30 μM Ciliobrevin D (Fig 2A), an inhibitor of the dynein ATPase motor. After 24 hours, there was a loss of primary cilia, replaced by a disorganized polymerization of AcTub (Fig 2A, Right Column), reminiscent of the tubulin arrangement in hypoxia-treated neuronal cell lines. Given that ciliobrevin D disrupted primary cilia, we established a dose-toxicity curve of Ciliobrevin D with N1E-115 cultures (Fig 2B). Ciliobrevin D became toxic to N1E-115 cells at concentrations of 10 μM and above (Fig 2B, *p<0.05 and **p<0.01). Immunohistochemistry for AcTub revealed that 30 μM concentrations of ciliobrevin D caused the loss of neurites and cilia in N1E-115 (Fig 2C).

In order to determine if cilia disruption sensitizes cells to apoptotic stimuli, N1E-115 cells were pretreated overnight with minimally toxic concentrations of 1 μM or 10 μM Ciliobrevin D. These cultures were then exposed to 8 hours of hypoxia, which was sublethal to DMSO treated controls. Both 1 μM and 10 μM Ciliobrevin D treated cultures showed a significant increase in cell death following 8 hours of hypoxia compared to controls (Fig 2D, **p< 0.01) indicating that the loss of cilia increased neuronal sensitivity to apoptosis.

**Fig 3:**
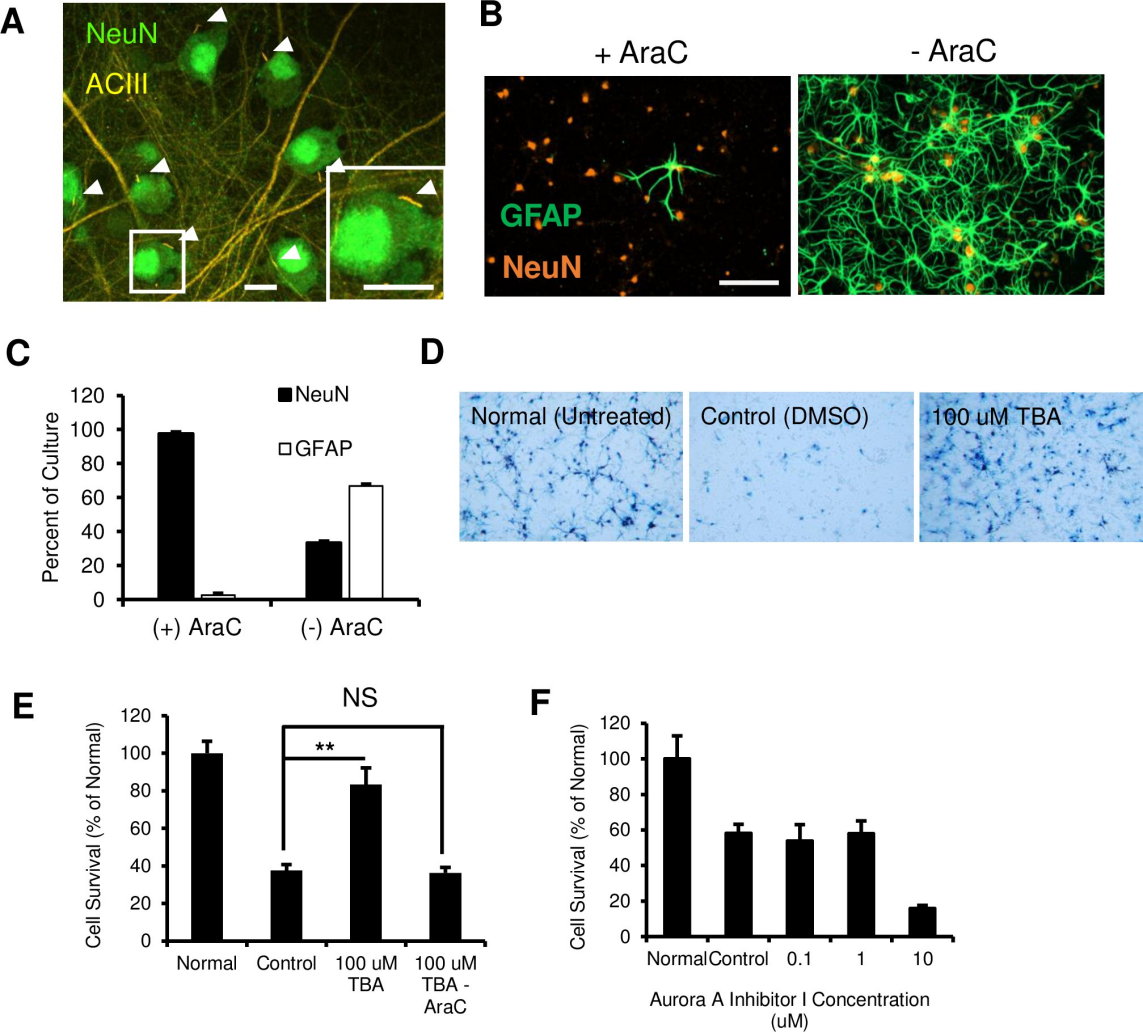
Tubastatin A, but not Aurora A Inhibitor, protects purified primary cortical neuron cultures from etoposide. (A) Micrograph showing cultured primary cortical neurons are ciliated (arrowheads). Scale bars = 10 μm. (B) Representative images of primary cortical neuron cultures with and without AraC treatment showing proliferation of glial cells in the culture without treatment. Scale Bar = 100 μm. (C) Quantification of cell cultures with or without AraC treatment, showing a significantly higher proportion of GFAP-positive cells in cultures without AraC purification. Data presented as mean ± SEM. (D) Representative images of MTT crystallization in surviving cortical neurons after etoposide toxicity, in normal (untreated), DMSO control, or TBA treated cultures. (E) TBA treatment significantly increased cell survival after etoposide insult compared to DMSO control, but not in cultures that did not receive AraC. (F) Quantification of cortical neuron survival after AurA inhibitor I treatment showing increased cell death after etoposide injury.

Next, we evaluated whether preventing cilia resorption could prevent neuron degeneration. To this end, we cultured primary rat cortical neurons that harbour cilia upon maturation (Fig 3A). Primary cilia in cortical neurons were visualized with immunofluorescence against Adenylyl Cyclase III (ACIII) [29]. We observed that without any purification steps, GFAP immunopositive cells outnumbered neurons in our cultures (Fig 3B and 3C), so glial contamination was eliminated by treating cultures with AraC. Etoposide, an inhibitor of topoisomerase, was used to injure neurons because it has been shown to cause cell-cycle re-entry in rat cortical neurons [30]. Moreover, Di Giovanni et al. (2005) found that CDK inhibitors flavopiridol, roscovitine, and olomoucine reduced cell death following etoposide treatment *in-vitro*, validating the compound as an inducer of ACC.

To interfere with cilia retraction, we utilized Tubastatin A (TBA), an inhibitor of HDAC6, and an Aurora Kinase A Inhibitor I (AAi). AAi is 1000 fold more selective for AurA than Aurora kinase B [31]. Tubastatin A is highly selective for HDAC6 with a 1000 fold greater selectivity against other isoforms with the exception of HDAC8 where it has a 57 fold greater selectivity [32]. TBA has been shown to restore cilia expression in cholangiocarcinoma [33] and prevent the deciliation of RPE1 by HDAC6 overexpression [34]. Pre-treatment of cortical neuron cultures with 100 μM TBA resulted in a significant increase in survival from etoposide induced apoptosis, compared to DMSO controls as measured by the MTT assay (Fig 3D and 3E, **p<0.01). Interestingly, the neuroprotective effects of TBA were abolished in neuron cultures that were not purified of glial cells (Fig 3E). This suggests that TBA was conferring neuroprotection through cell cycle inhibition rather than extra-ciliary neuronal tubulin stabilization, because it was cytotoxic to dividing glial cells. In contrast, although AurA inhibition stabilizes cilia from serum induced resorption [12], AAi was not neuroprotective for cultured rat cortical neurons (Fig 3F).

### Optic nerve transection induces abortive cell cycling and ciliary shortening in retinal ganglion cells

**Fig 4:**
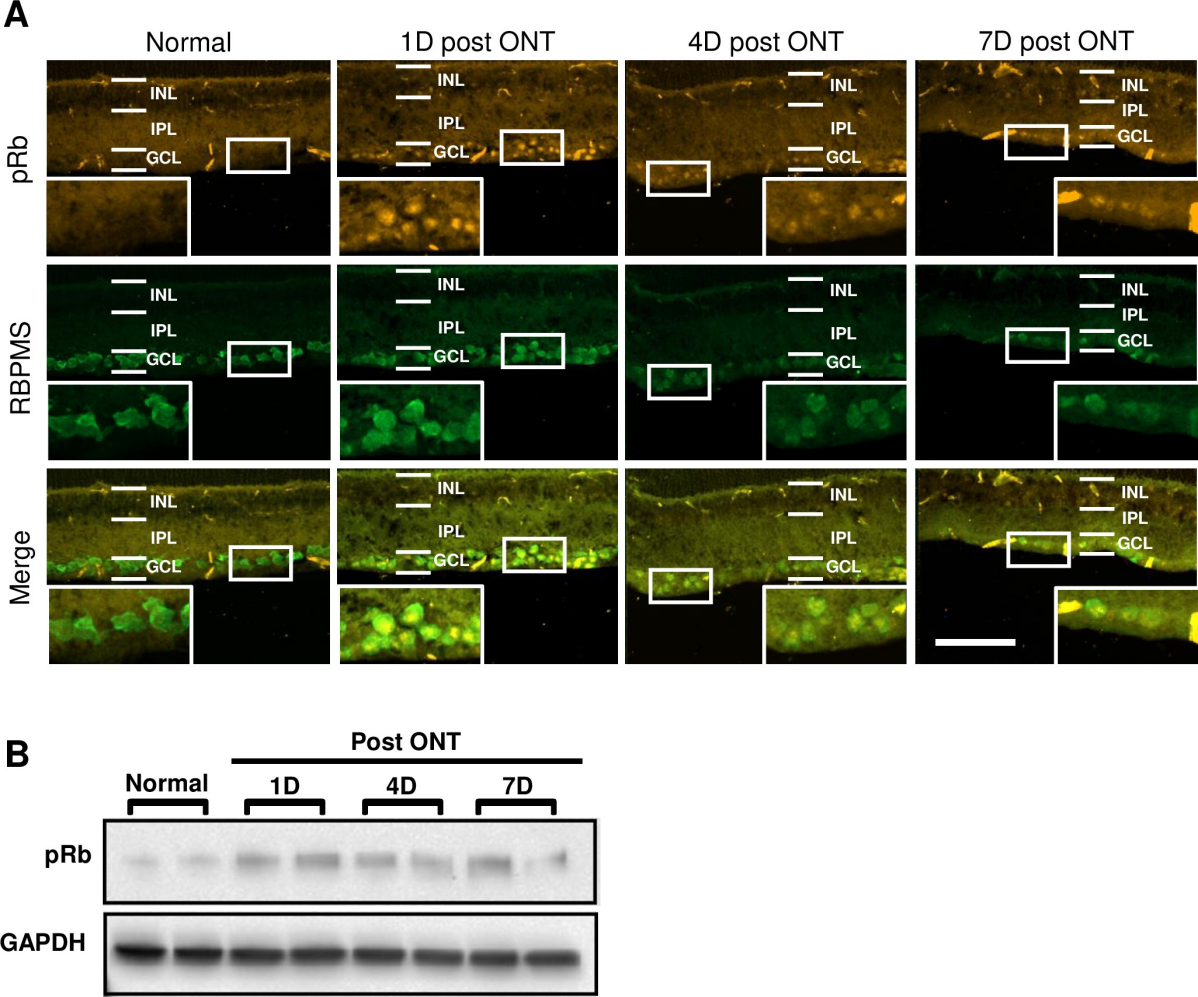
ONT induces pRb expression. (A) Immunofluorescent micrographs of transverse sections of the retina showing nuclear pRb expression in RGCs after ONT, at 1, 4, or 7 days postaxotomy. Scale bar = 100 μm. (B) Western blot of whole retinal lysates demonstrating elevated levels of pRb in transected retinas. Each band represents a whole retina.

We further investigated the relationship between primary cilia and ACC *in vivo* using a CNS injury model of optic nerve transection (ONT) in adult rats. ONT results in the apoptotic degeneration of 90% of retinal ganglion cells (RGCs) within 14 days after axotomy [35]. First, we sought to validate ONT as an appropriate model of neuronal ACC. We probed for pRb expression in RGCs after ONT as this cell cycle protein is observed in a number of human and experimental models of CNS pathologies [25,36–40]. Furthermore, pRb is a critical marker of cell division, initiating cell cycle re-entry [41]. At 1, 4, and 7 days after ONT, retinas were cryosectioned and the RGCs were identified by probing for RNA-binding protein with multiple splicing (RBPMS). Naïve rats presented no pRb expression in the RGCs whereas RGCs at 1, 4, and 7 days postaxotomy showed salient nuclear staining for pRb (Fig 4A), confirming that ONT was inducing cell cycling. As pRb expression was limited to the ganglion cell layer (GCL) we carried out Western blot analysis of whole retinal lysates and similarly found pRb elevation at 1, 4, and 7 days post ONT (Fig 4B).

**Fig 5:**
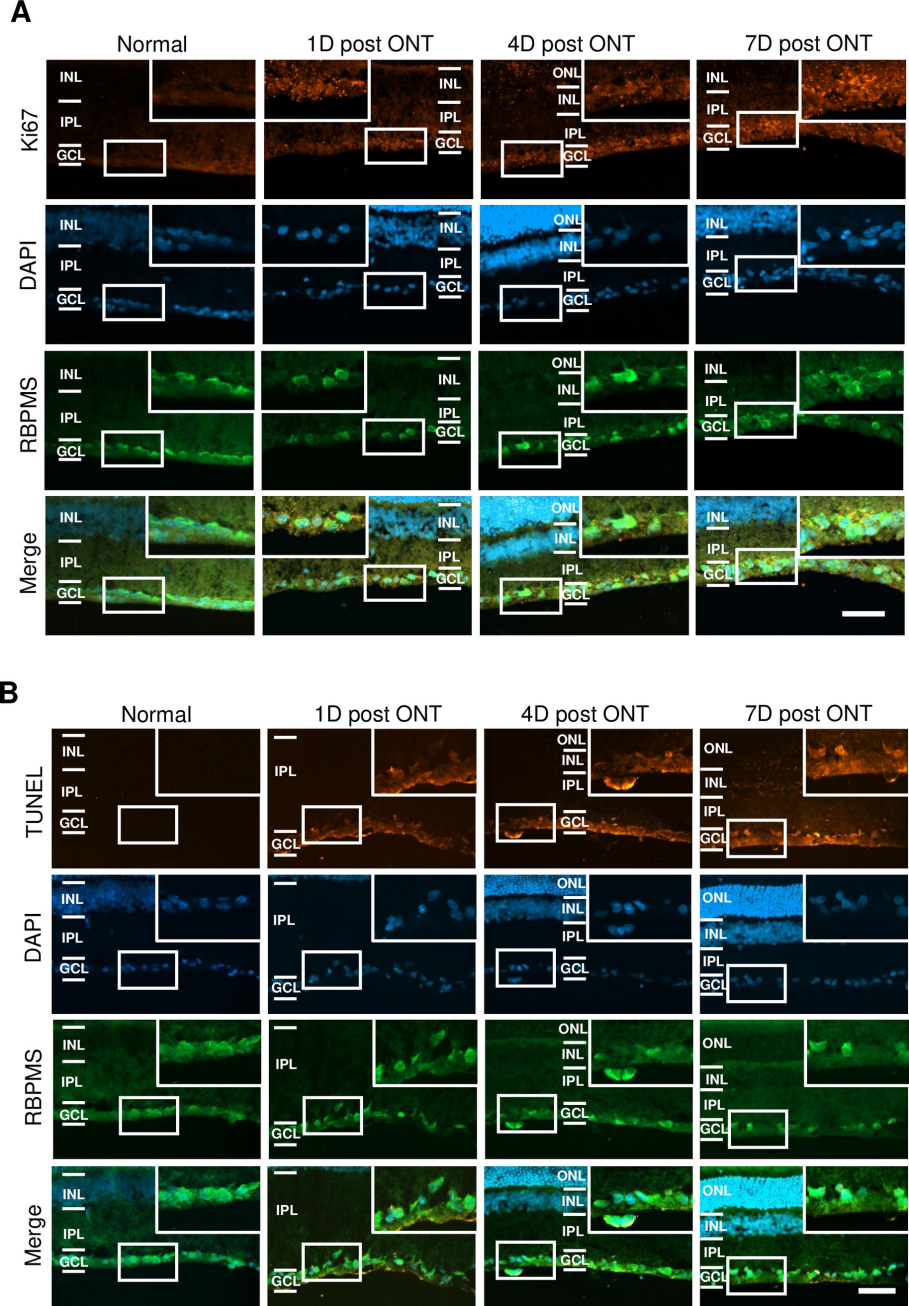
ONT induces Ki67 expression and DNA damage in RGCs. (A) Immunofluorescent micrographs of transverse sections of the retina showing diffuse Ki67 expression in the GCL after ONT, at 1, 4, or 7 days postaxotomy. Scale bar = 50 μm. (B) Immunofluorescent micrographs of transverse sections of the retina showing TUNEL expression in the RGCs after ONT, at 1, 4, or 7 days postaxotomy. Scale bar = 50 μm.

To complement the pRb expression, we probed for Ki67, which is detected in all phases of the cell cycle and is reported to be present during ACC [26,38,42]. We found positive Ki67 immunoreactivity in the GCL of axotomized retinas (Fig 5A). Furthermore, we also detected DNA fragmentation by TUNEL assay in axotomized RGCs (Fig 5B) which confirmed that apoptosis was occurring concurrently with the cell cycling.

**Fig 6:**
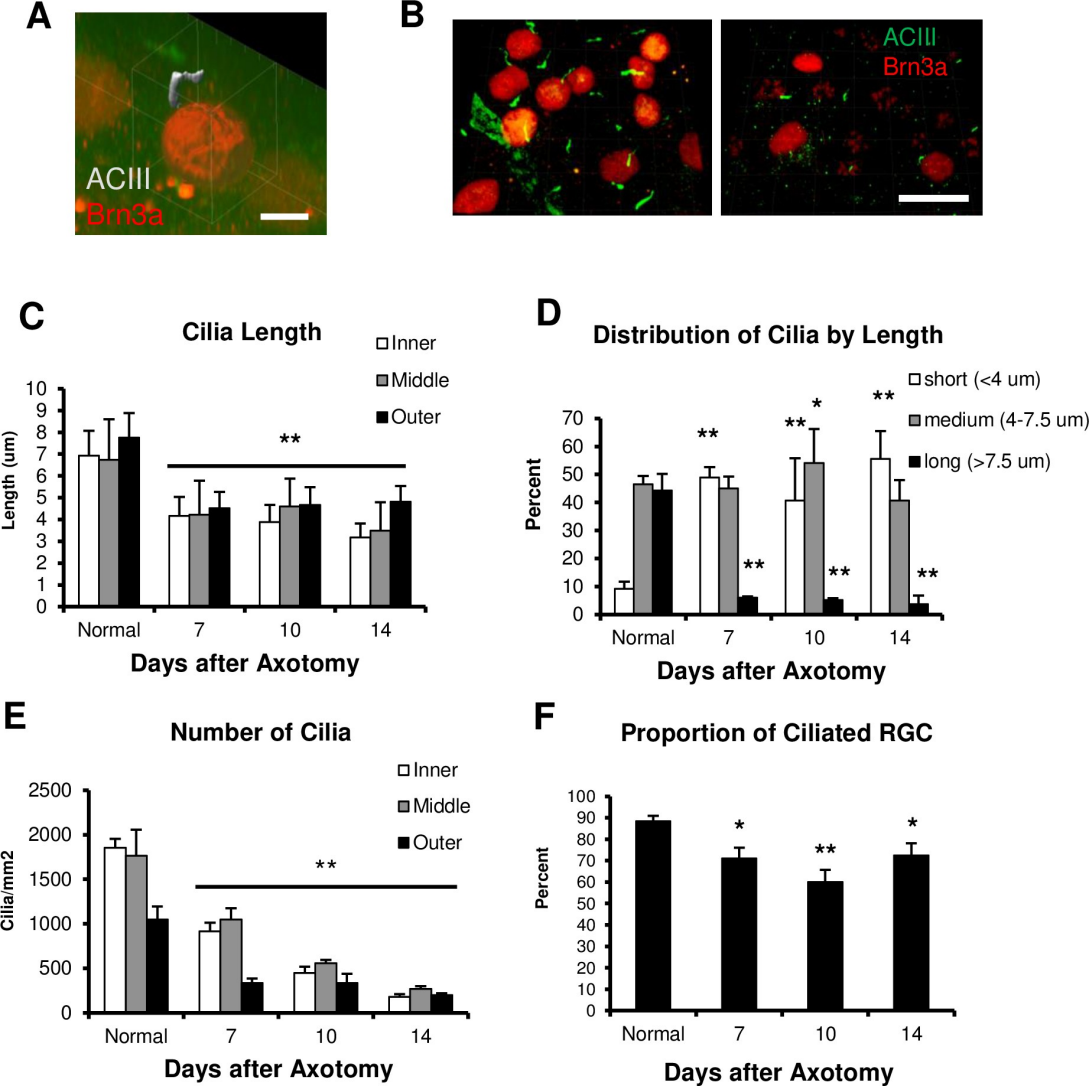
ONT induces ciliary shortening in RGCs. (A) Representative image of 3D voxel generation of an immunostaining for cilia (gray) and RGC nuclei (red) used in cilia quantification. (B) Left: Representative image of an immunofluorescent Z-stack of a naïve retina showing RGC nuclei (red) and cilia (green). Right: Representative image of an immunofluorescent Z-stack of the retina at 14 days post ONT. Scale bar = 20 μM. (C) The mean length of RGC primary cilia (μM) ± SEM, before and after ONT. **p<0.01. (D) Quantification of RGC cilia in normal and axotomized retinas, stratified by length. ONT induced a significant increase in the proportion of short cilia and a drastic decrease in the proportion of long cilia at each time point after ONT. **p<0.01. *p<0.05. (E) The average number of RGC primary cilia ± SEM quantified before and after ONT. **p<0.01. (F) The average percentage of ciliated RGCs ± SEM. Ciliated RGCs were significantly reduced after axotomy indicating complete loss of cilia in some RGCs following injury (*p<0.05, **p<0.01). A total of >50 cilia were measured for each group, n = 3 retinas per group.

After confirming that axotomized RGCs underwent ACC, we hypothesized that there would be shortening or complete loss of RGC cilia following ONT. To accurately quantify primary cilia length, the cilia were visualized in three-dimensional Z-stacks using confocal microscopy (Fig 6A). RGCs were identified using Brn3α, a nuclear marker that labels over 90% of RGCs [43]. Using 3D-reconstructions of the GCL, we quantified RGC cilia lengths from naïve retinas as well as retinas at 7, 10, and 14 days post-ONT. We first assessed the overall lengths of the cilia from the different eccentricities of the retina (inner, middle, outer), at the specified time points (Fig 6C). There were significant reductions in cilia lengths after axotomy at all retinal eccentricities; axotomized RGCs harboured cilia that were approximately half the length of those found in normal RGCs (Fig 6C, **p<0.01). To better understand changes in cilia length after ONT, cilia were stratified into short (<4 μm), medium (4–7.5 μm), and long groups (>7.5μm) (Fig 6D). This revealed continuous changes in the proportion of cilia length at all measured time points after axotomy. This revealed a drastic decrease in the fraction of long cilia together with an increase in the proportion of short cilia (Fig 6D, **p<0.01). The proportion of medium length cilia remained stable at 7 days after axotomy; however, there was significant increase in their proportion at 10 days post axotomy, possibly due to the continued disappearance of short cilia by complete resorption (Fig 6D, *p<0.05).

As expected, the number of RGC cilia decreased in a time course that correlated with RGC death after ONT [35], with a significant reduction in cilia counts at 7 days post ONT that continued until 14 days (Fig 6E, **p<0.01). Moreover, the percentage of ciliated RGCs significantly decreased after ONT (Fig 6F, *p<0.05, **p<0.01). In normal retinas, 88.5 ± 2.5% of RGC cilia could be accounted for, but this decreased to 71.0 ± 5% at 7 days post axotomy (**p<0.01), and further decreased to 60 ± 5.7% at 10 days (*p<0.05). Interestingly, there was an increase in RGC ciliation at 14 days after injury. This may be attributable to the deceleration in RGC death at 2 weeks post ONT and the extended survival of the remaining RGCs [35,44] resulting in a higher proportion of ciliated cells that survived ACC.

### Modulation of cilia resorption impacts retinal ganglion cell susceptibility to apoptosis

**Fig 7:**
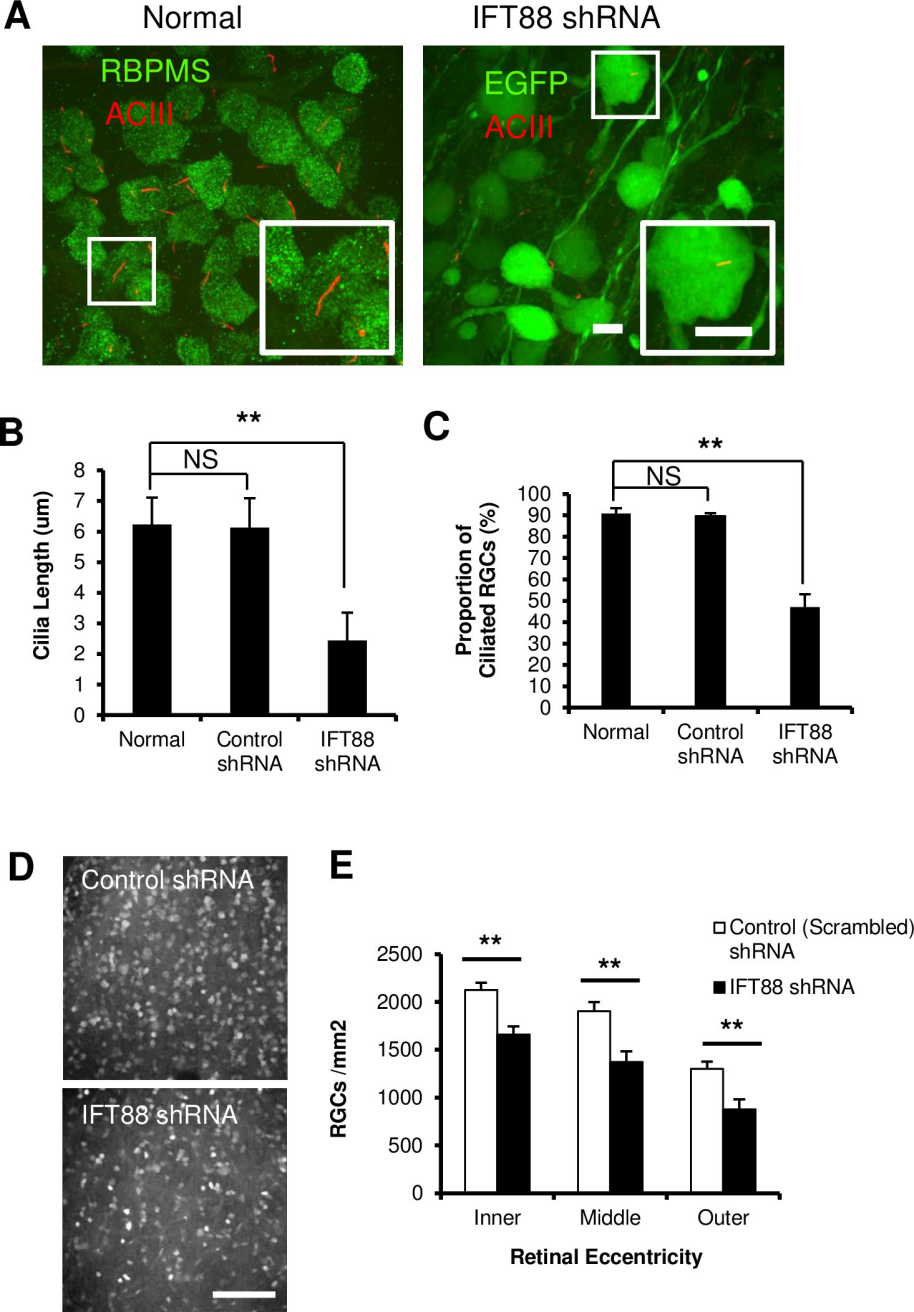
IFT88 shRNA shortens cilia and enhances RGC degeneration following ONT. (A) Left: Micrographs of flatmounted retinas showing ACIII immunolabeling of primary cilia in RBPMS-positive RGCs. Right: Micrographs showing GFP positive RGCs expressing AAV2 vectors encoding IFT88 shRNA. Scale bars = 10 μm. (B and C) The length of RGC cilia (B) and the proportion of ciliated RGCs (C) decreased at 3 weeks after AAV2-IFT88-shRNA vector injection. **p<0.01. (D) Representative epifluorescence micrographs of RBPMS immunolabeled RGCs in the inner eccentricity of flatmounted retinas at 7 days post-ONT, expressing control shRNA (top) or IFT88 shRNA (bottom). Scale bar = 100 μm. (E) Quantification of RGCs showing that IFT88 shRNA expression significantly decrease RGC survival at 7 days post ONT, relative to scrambled control hairpin. **p<0.01, n = 4 retinas.

Having demonstrated that injured RGCs simultaneously underwent cell cycling and cilia resorption, we aimed to link the two phenomena by manipulating cilia stability and examining its effect on neuronal survival. First, we targeted the cilia specific protein IFT88 for knockdown. IFT88 is a protein subunit of the intraflagellar complex B and its mutation results in the inability to form cilia [45,46]. We stably transduced RGCs with shRNA encoding viral vectors via intraocular injection of Adeno-Associated Virus 2 (AAV2) (>4 x 10^8^ GC per retina) (Fig 7A, Right). Confocal Z-stacks revealed that 3 weeks post viral injection, EGFP- IFT88 shRNA positive cells exhibited significantly shorter cilia (2.4 ± 0.9 um) compared to normal and control shRNA treated RGCs (Fig 7B, 5.6 ± 1.0 um and 6.1 ± 1.0 um, **p<0.01). Furthermore, the percentage of ciliated cells significantly dropped in IFT88 shRNA treated retinas (47.1 ± 6.0%) compared to normal and control retinas (90.9 ± 2.5% and 89.7 ± 1.3%, Fig 7C, **p<0.01). ONT was then carried out in rats treated with IFT88 shRNA encoding vectors. Compared to control shRNA, IFT88 shRNA-expressing retinas showed significantly reduced RGC survival in all eccentricities of the retina, at 7 days post-axotomy (Fig 7D and 7E, **p<0.01).

**Fig 8:**
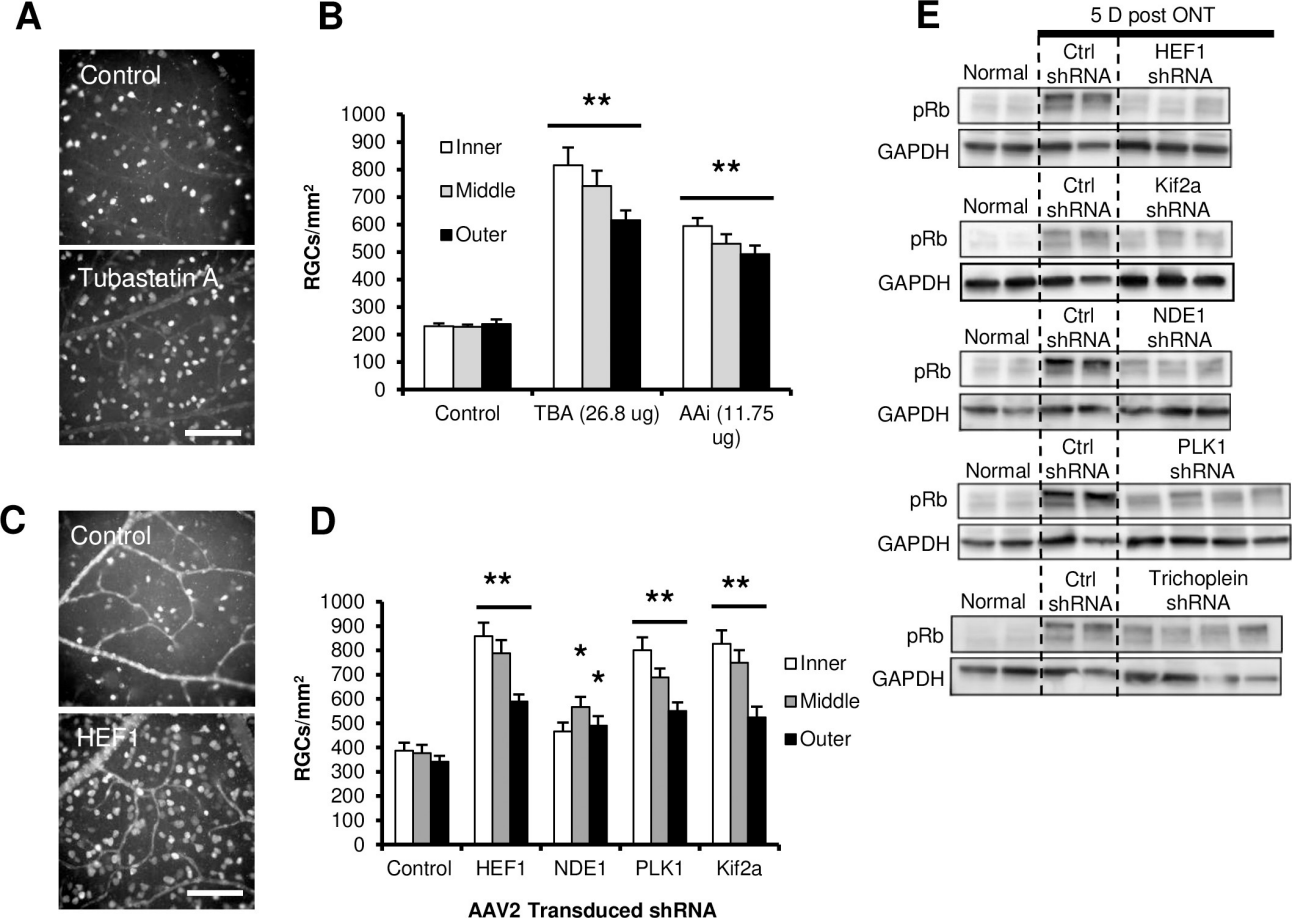
Inhibition of cilia disassembly protects RGCs and suppresses cell cycling after ONT. (A) Representative epifluorescence micrographs showing RBPMS immunolabeled RGCs in the inner eccentricity of flatmounted retinas at 14 days post-ONT, with DMSO (top) or TBA treatment (bottom). Scale bar = 100 μm. (B) Quantification of RGCs after intravitreal injection of the TBA (n = 4 retinas) or AurA inhibitor I (n = 5 retinas), demonstrating significantly increased RGC survival at 14 days post-ONT compared to DMSO control treatment (**p<0.01). (C) Epifluorescence micrographs showing RBPMS immunolabled RGCs in the middle eccentricity of flatmounted retinas at 14 days post-ONT, after control (scrambled shRNA) or AAV-HEF1-shRNA delivery. Scale bar = 100 μm. (D) Quantification of RGC survival at 14 days post-ONT, with AAV2 mediated expression of shRNAs that target HEF1, NDE1, PLK1, or Kif2a. ShRNAs significantly enhanced the survival of axotomized RGCs compared to scrambled control (n = 4 retinas per treatment) (**p<0.01, *p<0.05). (E) Western blots of whole retinal lysates from normal or 5 days post-ONT retinas with each band representing a whole retina. ShRNAs against HEF1, Kif2a, NDE1, PLK1, or TCHP decreased the levels of pRb compared to scrambled control shRNA.

As ciliary truncation exacerbated RGC death after ONT, we tested the opposite possibility, that stabilizing cilia by pharmacological inhibition of cilia disassembly proteins could promote RGC survival. TBA (6.8μg/μL) or AAi (2.9 μg/μL) diluted in DMSO were injected separately into the vitreous chamber at 3 and 7 days post ONT. Retinas were extracted 2 weeks post ONT and RGC survival was quantified by RBPMS immunolabeling [24]. There was a significant increase in RGC survival after TBA or AAi treatment, when quantified at 14 days after axotomy (Fig 8A and 8B, **p<0.01).

Building on these findings, we targeted additional cilia disassembly proteins for AAV-mediated knockdown by shRNA. Knockdown of HEF1, PLK1, or Kif2a resulted in significant improvement in RGC survival across all eccentricities of the retina (Fig 8C and 8D, **p<0.01), compared to retinas that received control (scrambled) shRNA expressing viral vectors. Additionally, NDE1 knockdown resulted in significant increase in RGC in middle and outer eccentricities (Fig 8D, *p<0.05) compared to controls.

Finally, we examined the expression of pRb by Western blot analysis in AAV-shRNA treated retinas at 4 days following ONT. The elevated levels of pRb that were found in control shRNA expressing retinas were reduced in retinas expressing shRNAs against HEF1, Kif2a, NDE1, PLK1, or Trichoplein (Fig 8E). Collectively, these findings show that cilia disassembly plays a role in initiating abortive cell cycle re-entry, and interfering with the ciliary resorption process can attenuate cell cycling and neuronal apoptosis in adult mammalian CNS neurons.

## Discussion

The involvement of cell cycle re-entry in neuronal apoptosis has been demonstrated in animal models and post-mortem examinations of the human CNS [5]. The primary cilia are highly relevant in this regard, as they are recognized to be a gatekeeper of the cell cycle by sequestering the centrioles at the cell membrane [21]. To our knowledge, this is the first demonstration that neuronal injury causes primary cilia shortening and that inhibiting the cilia disassembly mechanism reduces cell cycle re-entry. Thus, we address the disconnect that exists between the knowledge of ciliary control of cell cycle and the observation of cell cycle re-entry in neuronal diseases and injuries.

We found that both hypoxia and ONT induced cilia shortening in neuronal cell lines and RGCs respectively. In concert with previous studies showing that ACC is initiated in other models of neuronal apoptosis, this observation highlights a connection between cilia resorption and the initiation of proliferation signalling [26,47]. Furthermore, the increased metabolic activity of N1E-115 cells that was observed at 5 hours of hypoxic stress corresponds with the energy intensive nature of cell cycle activation. When the cells were returned to normoxia and metabolic activity normalized, there was a concurrent re-expression of primary cilia indicating a possible reversal of cell cycle initiation and rescue of the surviving cells. Additionally, we demonstrated that shortening primary cilia enhanced apoptosis, as it sensitized cultured neurons to hypoxia and accelerated the degeneration of RGCs after ONT. This presumably occurs through accelerated entry into ACC because it has been shown that longer cilia delay cell cycle progression [17].

In contrast to forcibly shortening cilia, pharmacological inhibition of HDAC6 protected cortical neuron cultures from etoposide induced apoptosis; however, only neuronal cultures that were purified of glial contamination were conferred this benefit, indicating that TBA was acting through cell cycle inhibition and toxic to other mitotic cells. By contrast, AurA inhibition enhanced cortical neuron death. This may have been attributable to the early role of AurA in neurite elongation by activating nuclear distribution protein nudE-like1 [48]. AurA is abundantly expressed in young post-mitotic neurons and its inhibition may have negatively impacted the growing cortical neuron culture, masking any protective effects of cilia stabilization. This highlights some of the protein function overlap between neurogenesis and cilia disassembly, likely related to the ability to reorganize tubulin, and the importance of inactivation upon neuronal maturation. HDAC6 and AurA inhibition along with shRNA knockdown of HEF1, NDE1, PLK1, or Kif2a was neuroprotective to RGCs after ONT. Furthermore, these treatments, along with TCHP knockdown, reduced the levels of pRb expression in RGCs after ONT, demonstrating that the neuroprotection was occurring through primary cilia-mediated suppression of ACC. The reduction of pRb levels is a crucial indication of cell cycle cessation as pRb signifies the overcoming of the restriction point where the cell is irreversibly committed to proliferation [49]. This reduction of pRb complements previous findings that the activity of cilia disassembly proteins are elevated in cell cycle progression but their knockdown or inhibition abrogates proliferation [13,17,50–52].

Additional proteins involved in cilia disassembly that were not examined in our study but deserve mention are Pitchfork (Pifo) and Tctex. Pifo activates AurA and is concentrated at the basal body of mouse embryonic nodal cilia at the early stages of cilia disassembly [53]. Tctex-1 is a light chain component of dynein that activates local F-actin polymerization to trigger endocytosis of the ciliary pocket and accelerate cilia resorption [54]. Haploinsufficiency of Pifo results in defects in cilia disassembly [53] while shRNA knockdown of Tctex-1 impairs cilia resorption, causing premature differentiation of neural progenitor cells [55]. Targeting these proteins in neuronal apoptosis may be of interest for future studies on ACC.

The commonality of ACC is evidenced by its observation in animal models of Alzheimer’s, Huntington’s, and Parkinson’s disease as well as stroke and traumatic brain injury [36,56–59]. Moreover, mitogenic markers such as Proliferating Cell Nuclear Antigen, pRb, cyclins, and CDKs have been found in CNS samples of Alzheimer’s and Parkinson’s disease patients while Huntington’s disease and ALS patients demonstrated elevated CNS levels of pRB and cyclin D1. This highlights the importance of investigating cell cycle control mechanisms that are applicable to multiple CNS degeneration models in an effort to develop therapeutic strategies for these incurable diseases. To this end, the ubiquity of primary cilia amongst neurons and its broad control over the cell cycle makes cilia an ideal target for preventing neuronal pathology in adult CNS.

Our study has focused on the disassembly component of the cilia but the mechanism by which the resorption is relayed to the cytoplasm or nucleus is not fully understood [60]. Ciliary control of the cell cycle is suggested to occur through the sequestering of cell cycle regulators within the organelle. Thus, the retraction of the cilia releases these molecular cues to the nucleus to initiate cell cycling [60,61]. This may occur by the way of Wnt and Hh signalling pathways that are modulated by the cilia [60,61]. The activation of the canonical Wnt pathway results in the accumulation of β catenin which activates the transcription of Cyclin D [62]. Jouberin (Jbn) associates with β catenin to prevent its proteasomal degradation and helps to translocate it to the nucleus [63]. Lancaster et al. (2011) demonstrated that in mouse embryonic fibroblast (MEF), primary cilia sequester Jbn away from the cell body by IFT and the disruption of retrograde IFT caused the accumulation of Jbn at the distal tip of the cilia. Additionally, inducing biciliation in MEF cells decreased nuclear β catenin and the Wnt response [64]. This suggests that the disassembly of the cilia releases additional Jbn into the cytosol to increase β catenin-dependent activation of cell cycling [61,64].

Hh signalling occurs through its surface receptors, Patched (Ptc), and its Hh effectors, Gli1-3 transcription factors that are localized inside the cilium [65]. These effectors activate genes involved in proliferation and metastasis [66]. In the absence of Hh, Gli2 is inactive and Gli3 is processed into its repressor form Gli3R in the cilia [67]. Upon Hh stimulation, Ptc relieves its inhibition of Smo which translocates to the cilium to prevent Gli3R processing. This activates Gli2 to promote the transcription of Gli1 [65,68,69]. Gli1-3 then promote the transcription of Cyclin D, Cyclin E, and E2F1 to advance G1/S transition [70]. As both Gli and Jbn are concentrated in the cilia, [65] the deconstruction of the organelle by HDAC6 and its activators may release these factors to the nucleus to initiate proliferation [61]. Further elucidating this mechanism presents the next step in understanding ciliary regulation of ACC. In conclusion, our study further erodes the long-held dogma that primary cilia are vestigial organelles by demonstrating their importance in maintaining neuronal quiescence and regulating programmed cell death in the adult mammalian CNS.
